# Efficacy and safety of ultrasound-guided intrathyroidal injection of glucocorticoids versus routine oral administration of glucocorticoids for subacute thyroiditis: Protocol of systematic review and meta-analysis

**DOI:** 10.1097/MD.0000000000018564

**Published:** 2019-12-27

**Authors:** Jinyan Li, Ji Zhang, Li Jiang, Ziling Li, Fang Li, Huixia Chen, Ling Feng

**Affiliations:** a Department of Ultrasound, Inner Mongolia Baogang Hospital (The Third Affiliated Hospital of Inner Mongolia Medical University), Baotou, Inner Mongolia Autonomous Region; b Department of Ultrasound, Beijing Ditan Hospital, Capital Medical University, Beijing; c Department of Endocrinology, Inner Mongolia Baogang Hospital (The Third Affiliated Hospital of Inner Mongolia Medical University), Baotou, Inner Mongolia Autonomous Region, China.

**Keywords:** efficacy and safety, hormone, intrathyroidal injection, protocol for a systematic review and meta-analysis, randomized control trial, subacute thyroiditis, ultrasound-guided

## Abstract

**Background:**

This study was designed to systematically evaluate the clinical efficacy and safety of ultrasound-guided intrathyroidal injection of glucocorticoids (GCs) versus routine oral administration of GCs for subacute thyroiditis (SAT) and to help seek evidence of evidence-based medicine (EBM) for ultrasound-guided intrathyroidal injection of GCs in the treatment of SAT.

**Methods:**

Seven Chinese and English databases, including Chinese National Knowledge Infrastructure, Wanfang Data, VIP Information China Science and Technology Journal Database, SinoMed, PubMed, Cochrane Library, and Embase, were searched to collect randomized control trials on ultrasound-guided intrathyroidal injection of GCs in the treatment of SAT, which were published up to July 1, 2019. According to the method as described in Cochrane Reviewers’ Handbook 5.1.0, the Cochrane Collaboration's tool for assessing risk of bias was employed to evaluate the quality of the literatures included. Statistical analysis was made by using Stata 12.0. The “metanif” command was used for sensitivity analysis to assess the stability of the results. Funnel diagram method, Egger linear regression method, and clipping complement method were used to evaluate publication bias.

**Results:**

This study was carried out in strict accordance with the standard procedures for meta-analysis in the Cochrane Reviewers’ Handbook 5.1.0. Critical data about the primary and secondary outcome measures were obtained by statistical analysis.

**Conclusion:**

This study would draw a definite conclusion about whether ultrasound-guided intrathyroidal injection of GCs is effective and safe in the treatment of SAT on the basis of EBM. This conclusion would provide scientific evidence for the clinical treatment of SAT.

## 1 Introduction

Subacute thyroiditis (SAT) is the most common painful thyroid disease. With the accelerating pace of life and the increasing work pressure in modern society in recent years, the incidence of SAT is on the rise year by year.^[1]^ According to statistical analysis, the incidence of SAT is 4.9/100,000 persons, accounting for 5% of all thyroid diseases. The occurrence of SAT changes with seasons markedly, and it is very likely to occur from July to October.^[2]^ SAT often occurs in 30 to 50 years old females. Due to its atypical clinical symptoms at the early stage, such disease is easy to be misdiagnosed or missed. Some literatures report that the misdiagnosis rate of SAT at the first visit is up to 15% to 50%.^[3]^ It is generally accepted in Western medicine that SAT is linked to viral infection and allergic reaction. Some study has shown that viral infection may lead to the destruction of thyroid follicular cells and the colloid released will cause foreign body reaction in the thyroid tissue and fibrosis.^[4]^ Some study also finds that the occurrence of SAT is associated with genetic factors.^[5]^ The course of SAT often lasts for 2 to 3 months, and typical SAT may be divided into 3 stages according to the clinical characteristics. At the acute stage, SAT causes severe pain, thyroid follicular cells are damaged, and thyroid hormones are released into blood, which may result in transient hyperthyroidism; at the remission stage transient hypothyroidism will occur; at the recovery stage, the thyroid function may recover to normal, but individual patients will suffer from permanent hypothyroidism.

Among the therapeutic strategies for SAT in clinical practice, oral administration of glucocorticoids (GCs) is still one of the commonly-used therapies.^[6]^ Although this method can improve the clinical symptoms of the patients, the adverse reactions (such as increased body weight, increased appetite, abdominal discomfort, insomnia, etc) associated with the systemic administration of GCs may occur. As a consequence, medication compliance is poor and some patients stop taking medication at the early stage, leading to incomplete drug treatment, unsatisfying overall curative efficacy, and a high recurrence rate.^[7]^ The recurrence rate of SAT patients treated with nonsteroidal anti-inflammatory drugs and GCs is still as high as 20% to 30%. A follow-up study of Japanese SAT patients treated with GCs (Arao et al) indicates that the time from decreasing the hormone dosage to 5 to 10 mg/d (maintenance dose) is negatively correlated with the recurrence rate, and it is necessary to extend the medication time to lower the recurrence rate.^[8]^ However, complications are increasing and become severer with the long-term use of GCs, especially for patients with hypertension, diabetics, and ulcer. The systemic administration of GCs may cause poor control of blood pressure and glucose, aggravate ulcer and increase such risks as cerebral hemorrhage and upper gastrointestinal hemorrhage among SAT patients. ^[9]^ Even so, the curative efficacy of GCs is definite in the treatment of SAT. Is it possible to achieve a better effect than oral administration of GCs and reduce the resulting adverse reactions by changing administration route? Therefore, by referring to the intrathyroidal injection of immunosuppressant or GCs for the treatment of autoimmune thyroiditis, some Chinese scholars treat SAT with the intrathyroidal injection of GCs and complete the whole process of puncture and drug injection under the guidance and monitoring of ultrasound.^[10]^ However, the majority of relevant literatures have small sample sizes, poor research quality, and inconsistent conclusions, which limits their values for clinical reference. Considering the above controversies, this study was carried out to evaluate the clinical efficacy and safety of ultrasound-guided injection of GCs for the treatment of SAT by meta-analytic methods, hoping to provide scientific theoretical evidence for the treatment of SAT in clinical practice.

## 2 Methods

### 2.1 Protocol and registration

A protocol had been registered for this study in PROSPERO (CRD42019147376). https://www.crd.york.ac.uk/PROSPERO.

### 2.2 Ethics and dissemination

Since this study is not a clinical trial, it is not necessary to seek approval from the ethnic committee as the same is done for clinical trials. The results of this study would be published in a high-quality international academic science citation index journals.

### 2.3 Eligibility criteria

#### 2.3.1 Types of studies

All randomized control trials (RCTs) on ultrasound-guided intrathyroidal injection of GCs for the treatment SAT in which “random allocation” is involved, regardless of whether the blind method and allocation concealment are mentioned, were included without limiting the language.

#### 2.3.2 Types of participants

Inclusion criteria:

(1) RCTs.
(2) Meeting the diagnostic criteria for SAT.
(3) During treatment, the observation group was intrathyroidal injected with GCs, while the control group orally took GCs. Confounding treatment factors affecting outcome measures (such as oral use of nonsteroidal anti-inflammatory drugs) were eliminated.
(4) The outcome measures of the studies included must contain the following: effective rate, clinical symptoms (fever, pain, and thyroid enlargement), remission time, adverse reactions, recurrence rate, and change of erythrocyte sedimentation rate (ESR).

If the research results were expressed in different ways, literatures with data that can be pooled through data conversion were included.

Exclusion criteria:

(1) Descriptive studies, reviews, and literatures without full text and case report available;
(2) Simple goiter;
(3) Patients with concurrent hyperthyroidism or thyroid malignancy, children and patients with gestational diabetes mellitus;
(4) Animal experiments and non-original literatures;
(5) Literatures with no complete data available;
(6) Literatures with repeatedly published research results;
(7) Literatures with data that cannot be pooled through data conversion.

#### 2.3.3 Types of interventions

Experimental group: ultrasound-guided intrathyroidal injection of GCs.

Control group: oral administration of GCs.

#### 2.3.4 Types of outcome measures

##### 2.3.4.1 Primary outcomes

(1) Remission time of fever;
(2) Remission time of local pain of thyroid;
(3) Remission time of thyroid enlargement;
(4) Clinical efficacy.

##### 2.3.4.2 Secondary outcomes

(1) Time to return normal ESR;
(2) Recurrence rate;
(3) Occurrence rate of adverse reaction;
(4) Thyroid function free triiodothyronine, free tetraiodothyronine, and thyroid-stimulating hormone;
(5) Serum inflammatory indicators: C-reactive protein and interleukin 6;
(6) Changes of serum antibodies: anti-thyroglobulin antibodies and thyroid microsomal antibody.

### 2.4 Search methods for the identification of studies

#### 2.4.1 Electronic searches

PubMed, Cochrane Library, and Embase (English databases), and Chinese National Knowledge Infrastructure (http://www.cnki.net), Wanfang Data (http://www.wanfangdata.com.cn), VIP Information China Science and Technology Journal Database (http://www.cqvip.com/), and SinoMed (http://www.sinomed.ac.cn) (Chinese databases) were searched by combining subject term and free term to collect papers published up to July 1, 2019.

#### 2.4.2 Other sources

Also, we will search relevant reviews, references of the literatures included and conference papers were retrieved manually to check if any literature meets the inclusion criteria to reduce omission-induced publication bias that may affect the reliability and stability of the conclusion

#### 2.4.3 Search strategy

The Chinese and English terms used were as follows: “ultrasound guidance,” “intrathyroidal injection,” “local injection,” “subacute thyroiditis,” “thyroiditis, subacute,” “glucocorticoids,” “dexamethasone,” “prednisone,” “betamethasone,” “limethason,” and “methylprednisolone;” “chaoshengyindao,” “jiazhuangxianneizhushe,” “jubuzhushe,” “yajixingjiazhuangxianyan,” “yajiayan,” “tangpizhijisu,” “disaimisong,” “ponisong,” “qiangdisong,” “beitamison,” “limeidasong,” and“jiaponilong.” These search terms were retrieved from article titles, keywords and abstracts. Table 1 shows the Search strategy for the PubMed database.

**Table 1:**
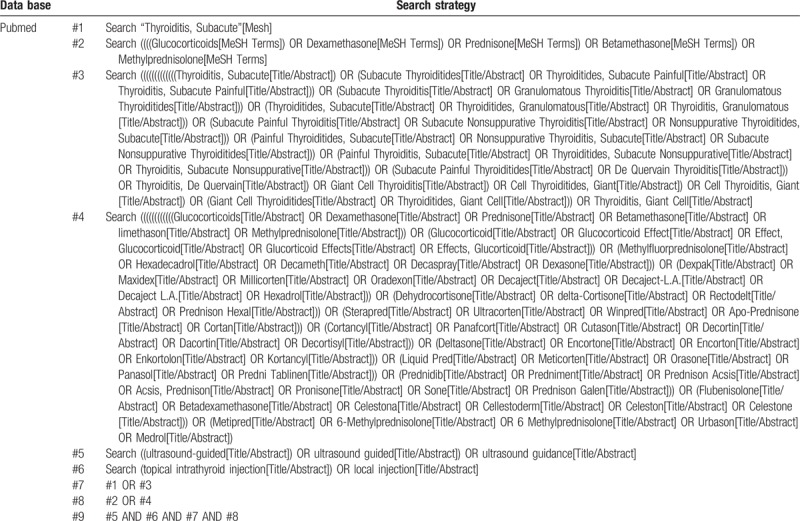
Search strategy for the PubMed database.

### 2.5 Data collection and analysis

#### 2.5.1 Data extraction

The 2 researchers independently searched and screened all the documents according to established standards.

**Figure 1:**
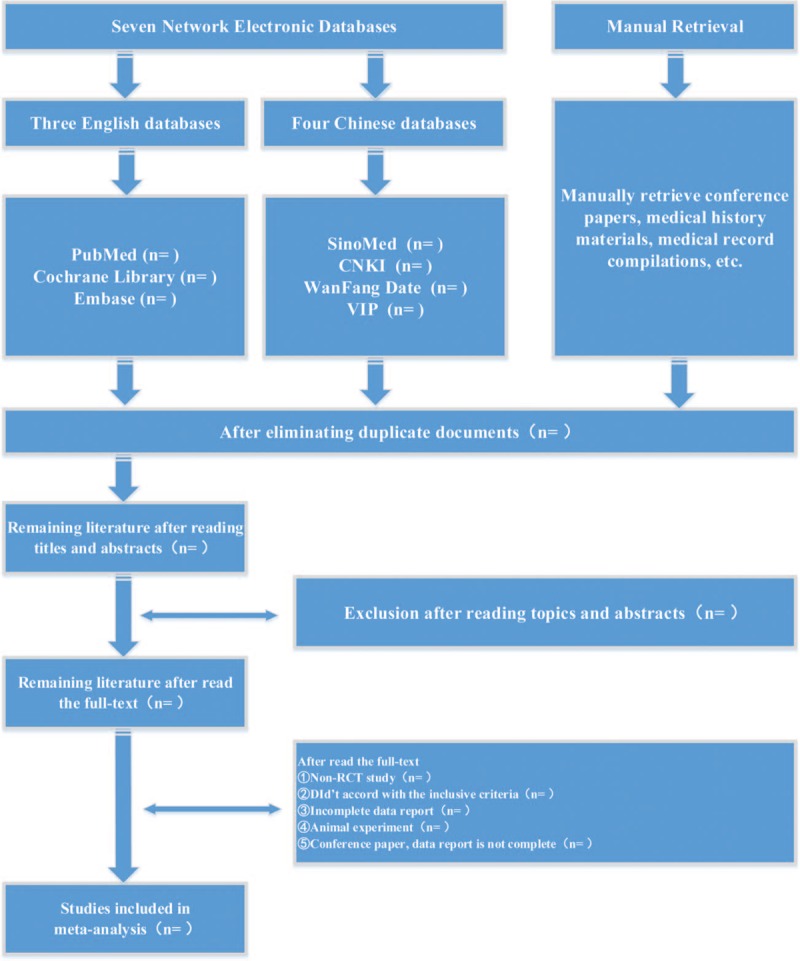
Flow chart of study selection.

Literature screening: relevant literature statistical table, information summary table, and literature quality evaluation table were prepared. Literatures searched in accordance with the search strategy were sorted out by 2 independent investigators according to the inclusion and exclusion criteria in the following steps:

(1) Preliminary screening: first, repeated literatures were ruled out, and titles, abstracts, and sources and types of literatures were read independently to eliminate obviously irrelevant literatures, such as reviews and animal experiments. Any literature with uncertainty was not eliminated temporarily until the full text was read through to make the final decision.
(2) Fine screening: the full texts of articles likely to be included were read to decide whether they meet the inclusion criteria. Literatures with incomplete data or disagreement were included temporarily, and 2 investigators cross-checked the screening results. If there was any disagreement, whether the literature involved should be included was decided through mutual discussion. A third person would help solve the disagreement where necessary. The screening process of the paper is shown in Figure 1.

Data extraction: the following information were extracted from each study included: The first author, nationality, year of publication, patients’ basic information, sample size and experimental grouping, experimental drugs and their dosages, duration of treatment, and target outcome (including primary outcome measures and secondary outcome measures).

#### 2.5.2 Addressing missing data or unclear measurement scales

For literatures with incomplete or unclear information, complete information was obtained by contacting the original authors.

#### 2.5.3 Assessment of heterogeneity

All the studies included in this meta-analysis were RCTs. As recommended by the Cochrane Reviewers’ Handbook 5.1.0, the Cochrane Collaboration's tool for assessing the risk of bias for RCT including 6 aspects was used to conduct a risk assessment on each study included in the meta-analysis.^[11]^ The results of the risk of bias assessment were divided into 3 types: low risk of bias, high risk of bias, and unknown risk of bias. The assessment involved in the following 6 aspects:

(1) Whether the generation of random sequence follows the principle of randomization;
(2) Whether the allocation scheme is concealed, that is to say, whether the research executers and subjects can predict the allocation result;
(3) Whether the research executors and objects are blinded.
(4) Whether the result data are complete;
(5) Whether the research results are reported selectively;
(6) Whether there is another source of bias.

Research quality was graded according to the result of the risk of bias assessment on the literatures included. Literature quality was divided into 3 grades: grade A, B, and C. Grade A: All the 6 criteria above were rated as low-risk, indicating the minimum possibility of various biases. Grade B: Only some of the 6 criteria above were rated as low risk, suggesting the medium possibility of various biases. Grade C: No low risk item in the 6 criteria above, indicating the high possibility of various biases. Risk of bias assessment on the literatures included was finished by 2 investigators independently and the results were cross-checked. Any inconsistency in the results would be solved through mutual discussion. Assistance would be sought from a third-person when necessary.

#### 2.5.4 Data analysis

Statistical analysis was made by using RevMan 5.3 (provided by Cochrane Collaboration). *Q* test was carried out to analyze the heterogeneity of the literatures included. If there was no statistically significant heterogeneity among these studies (*P* > .10 or *I* ^2^ < 50%), we could consider that there was homogeneity among multiple similar studies, and the combined effect size was calculated using a fixed-effect model. On the contrary, if there was heterogeneity (*P* < .10 or *I* ^2^ > 50%), it was required to analyze whether these studies have the same sources of heterogeneity (design scheme, measuring method, drug dose, route of drug administration, etc). For heterogeneity from these causes, subgroup analysis may be performed to merge statistics. Where clinical heterogeneity was not obvious and it is impossible to identify the source of remarkable statistical heterogeneity, a random-effects model was employed. For studies for which it is impossible to make meta-analysis or those with evident clinical or methodological heterogeneity or incomplete data, descriptive analysis was made. Dichotomous data were analyzed by using risk ratio and 95% confidence interval (CI). For continuous variable data, standardized mean difference and 95% CI were used to analyze and combine the effect sizes, and the results of the meta-analysis were displayed in a forest plot. Publication bias was quantified by the Egger linear regression method (*P* > .05 suggests no publication bias; *P* < .05 suggests possible publication bias). Furthermore, the nonparametric clipping complement method was employed to determine whether it is necessary to further include studies to reduce the publication bias of the research conclusion and to evaluate the stability of the combined statistical results.

#### 2.5.5 Sensitivity analysis

The so-called sensitivity analysis is a method of testing the stability of the target object. Sensitivity analysis involves in re-analysis after eliminating all the abnormal factors affecting the research activity, as well as the comparison between the results obtained and those without eliminating abnormal factors, thereby analyzing the influence of abnormal factors on the integration effect. If no fundamental difference is found in such comparison, the research result is considered to be relatively reliable. Otherwise, there are key factors affecting the effect of the whole research process, and it is required to be cautious about the conclusion. An in-depth analysis may be made to explore the source of these key factors.^[12]^

#### 2.5.6 Subgroup analysis and solutions to heterogeneity

According to different characteristics of the studies included, subgroup analysis was developed to investigate the factors that may affect the evaluation result. Representative characteristics were selected. The literatures included were divided into several subgroups before effect sizes were combined again for each subgroup. The consistency between the results of subgroup analysis and meta-analysis was observed to judge whether each factor has an important influence on the result of a combined effect, which is also of great significance for guiding individual processing.^[13]^ If the sample size of subgroup analysis is large enough, the result of subgroup analysis is reliable and can better support or overthrow the result of the combined effect of the meta-analysis.

#### 2.5.7 Assessment of reporting bias

Bias refers to the difference between corollary result and actual value that may be obtained at different phases of clinical trials, including selection bias, performance bias, measurement bias, follow-up bias, and reporting bias.^[14]^ The first 4 biases may be reduced by controlling the research quality of the literatures included. However, reporting bias is mainly evaluated based on the assessment of publication bias. The evaluation method was selected according to the quantity of the studies included. Stata14.0 was employed to make statistical analysis and draw diagrams. A qualitative evaluation was performed by the funnel-plot-based method when there were more than 7 studies. A qualitative evaluation was carried out by Egger linear regression method when there were less than 7 studies. *P* < .05 was considered to suggest publication bias. The stability of the result may be verified by clipping complement method.^[15]^

## 3 Discussion

The pathogenesis of SAT remains unknown, and the possible mechanisms include viral infection, immune disorder, and genetic factors. Most of the previous studies have shown that SAT is associated with a viral infection, especially Coxsackie virus, Echovirus, and other common pathogenic viruses. A viral infection will act on and destruct thyroid follicular cells, which leads to the release of colloidal substances from thyroid follicular cells and the chemotaxis of lymphocytes and multinuclear cells, resulting in foreign body reaction in local thyroid. A typical pathological change is the formation of giant cells around colloidal substances, finally leading to fibrosis.^[16]^ Virus or vaccine results in the occurrence and development of SAT the through immune mechanism, which confirms that local immune disorder of thyroid is an intermediate link of SAT from another perspective. Scholars Wang Xiaoling et al who have proven that the level of regulatory cells (Treg) is decreased in the presence of SAT, Treg, and other immune cells and cytokines participate in the occurrence and development of SAT, and anti-inflammation and immunological regulation may be the basis for treating SAT.^[17]^

GCs are a class of steroid hormones secreted by the fascicular cells of the adrenal cortex. They can synthesize special proteins by activating or regulating the gene transcriptions of cytokines and polypeptidase, thus inhibiting the production of inflammatory mediators and suppressing antigen-antibody reaction and other mechanisms. Hence, GCs have such pharmacological actions as anti-inflammation, antiviral action and immunosuppression.^[18]^ The mechanism of action of GCs in the treatment of SAT is that GCs can inhibit the chemotaxis and aggregation of neutrophil granulocytes and macrophage in local thyroid and prevent the formation of giant cells and granulation tissue. The thyroid is an important immune tissue in the human body, while GCs can regulate the immune function and restore the local immunological imbalance. With the rapid development of ultrasonics in recent years, the ultrasonic technique has made remarkable achievements in assisting diagnosis and treatment. For instance, the ultrasound-guided percutaneous biopsy and ablation allow real-time whole-process monitoring. Although intrathyroidal injection is an invasive procedure, it avoids excessive puncture depth and bypasses blood vessels under the guidance of ultrasound thanks to the ultrasound-guided fine-needle injection.[^19^,^20^] The advantages of ultrasound guidance are as follows: less injury in local tissue, a higher drug concentration in the lesion than oral administration, and easy operation; the whole process of puncturing and drug injection is completed under the guidance and monitoring of real-time ultrasound such that the puncture path can be selected precisely, the angle and depth of needle can be identified and totally controlled, and the needle can penetrate the target.^[21]^

Currently, there have been controversies over the clinical efficacy and safety of intrathyroidal injection of GCs for the treatment of SAT. Therefore, this study evaluates the efficacy and safety of intrathyroidal injection of GCs versus oral administration of GCs for the treatment of SAT by comprehensively searching relevant studies. In this study, we would draw a scientific conclusion and provide evidence of EBM for selecting the mode of drug administration in the clinical treatment of SAT.

## Author contributions

**Conceptualization:** Jinyan Li, Ling Feng.

**Data curation:** Jinyan Li, Ji Zhang.

**Investigation:** Ling Feng.

**Methodology:** Li Jiang, Ziling Li.

**Software:** Jinyan Li, Ji Zhang, Fang Li, Huixia Chen.

**Writing – original draft:** Jinyan Li, Ji Zhang, Li Jiang, Ziling Li, Fang Li, Huixia Chen.

**Writing – review and editing:** Ling Feng.

Ling Feng orcid: 0000-0001-7043-9498.
